# The Association of Genetic Markers Involved in Muscle Performance Responding to Lactate Levels during Physical Exercise Therapy by Nordic Walking in Patients with Long COVID Syndrome: A Nonrandomized Controlled Pilot Study

**DOI:** 10.3390/ijms25158305

**Published:** 2024-07-30

**Authors:** Ángel Lizcano-Álvarez, David Varillas-Delgado, Roberto Cano-de-la-Cuerda, Carmen Jiménez-Antona, Alberto Melián-Ortiz, Alberto Molero-Sánchez, Sofía Laguarta-Val

**Affiliations:** 1 Department of Nursing and Stomatology, Faculty of Health Sciences, Universidad Rey Juan Carlos, 28922 Madrid, Spain; angel.lizcano@urjc.es; 2 Exercise and Sport Science, Faculty of Health Sciences, Universidad Francisco de Vitoria, 28223 Pozuelo, Spain; 3 Department of Physical Therapy, Occupational Therapy, Rehabilitation and Physical Medicine, Faculty of Health Sciences, Universidad Rey Juan Carlos, 28922 Madrid, Spain; roberto.cano@urjc.es (R.C.-d.-l.-C.); carmen.jimenez@urjc.es (C.J.-A.); alberto.molero@urjc.es (A.M.-S.); sofia.laguarta@urjc.es (S.L.-V.); 4 Faculty of Nursing and Physiotherapy, Universidad Pontificia de Salamanca, 28015 Madrid, Spain; amelianor@upsa.es

**Keywords:** COVID-19, long COVID, genetics, physical therapy strategy, aerobic training, muscle response

## Abstract

Several genetic markers have shown associations with muscle performance and physical abilities, but the response to exercise therapy is still unknown. The aim of this study was to test the response of patients with long COVID through an aerobic physical therapy strategy by the Nordic walking program and how several genetic polymorphisms involved in muscle performance influence physical capabilities. Using a nonrandomized controlled pilot study, 29 patients who previously suffered from COVID-19 (long COVID = 13, COVID-19 = 16) performed a Nordic walking exercise therapy program for 12 sessions. The influence of the *ACE* (rs4646994), *ACTN3* (rs1815739), *AMPD1* (rs17602729), *CKM* (rs8111989), and *MLCK* (rs2849757 and rs2700352) polymorphisms, genotyped by using single nucleotide primer extension (SNPE) in lactic acid concentration was established with a three-way ANOVA (group × genotype × sessions). For *ACE* polymorphism, the main effect was lactic acid (*p* = 0.019). In *ACTN3* polymorphism, there were no main effects of lactic acid, group, or genotype. However, the posthoc analysis revealed that, in comparison with nonlong COVID, long COVID increased lactic acid concentrations in Nordic walking sessions in CT and TT genotypes (all *p* < 0.05). For *AMPD1* polymorphism, there were main effects of lactic acid, group, or genotype and lactic acid × genotype or lactic acid × group × genotype interactions (all *p* < 0.05). The posthoc analysis revealed that, in comparison with nonlong COVID, long COVID increased lactic acid concentrations in Nordic walking sessions in CC and CT genotypes (all *p* < 0.05). Physical therapy strategy through Nordic walking enhanced physical capabilities during aerobic exercise in post-COVID19 patients with different genotypes in *ACTN3* c.1729C>T and *AMPD1* c.34C>T polymorphisms. These findings suggest that individuals who reported long COVID who presumably exercised less beforehand appeared to be less able to exercise, based on lactate levels, and the effect of aerobic physical exercise enhanced physical capabilities conditioned by several genetic markers in long COVID patients.

## 1. Introduction

Long COVID has been defined in various ways, with the most common description being symptoms that persist for more than three months after the initial onset of symptoms [1]. Among the more than 200 clinical symptoms associated with long COVID, the most frequent are fatigue and dyspnea (shortness of breath) [2,3], affecting multiple organ systems, including the pulmonary and cardiovascular systems [3,4,5,6]. The primary clinical symptoms of long COVID syndrome include thrombotic events, brain fog, myocarditis, respiratory distress, fatigue, and muscle aches and pains. Additionally, risk factors such as age, high body mass index, female sex, previous hospitalization, and smoking have been identified [7].

Among the neuromuscular system symptoms experienced by individuals with long COVID include muscle weakness which can result from the direct effects of the virus on muscle tissues or from the body’s immune response and could impact daily activities and physical functioning [8,9]. Fatigue also is a common symptom in long COVID, and it can be related to neuromuscular issues [10]. The mechanisms of these neuromuscular symptoms are not fully understood, but it may be due to the ongoing immune response, disrupted sleep patterns, or other factors affecting muscle function as genetic factors and the condition can vary widely between subjects [11].

The role of physical exercise in the treatment of long COVID is a topic of active research. Some preliminary studies suggest that gradual, supervised exercise may have benefits for people experiencing long COVID [12,13,14,15], but it is important to emphasize that not all long COVID patients are in the same physical condition and cardiopulmonary system damage [4,16]. There are some key aspects to consider in relation to physical exercise in the context of long COVID: gradualness, medical supervision, types of exercise, and psychological support [17,18]. Although research is beginning to examine this new condition, serious questions remain about diagnostic identification, limiting the best therapeutic approach. However, it has been shown that aerobic physical exercise can improve pulmonary, cardiovascular, and neuromuscular function, although due to the individual characteristics of the patients, it is still necessary to know the characteristics of the effects of exercise-based treatments on long COVID-19 syndrome are required to give practical insights about what type of exercise should be preferably prescribed, with emphasis on intensity, load management, and adherence strategies [14] of both strength and endurance training [19].

The effects of long COVID on the oxidative capacity of patients are unclear, as is the production of the product showing this condition and the use of anabolic pathways during exercise, such as lactic acid, which may contribute to this chronic fatigue, compromised lung function, and cardiac dysfunction.

The individual characteristics of the patients are defined by their genetics, and through their knowledge, these individual training strategies could be adopted for subjects with long COVID syndrome. Genetics could play a key role in muscle and human performance [20]. There are several genetic factors that can affect muscle performance: muscle composition and fiber type, muscular strength, response to training, metabolism and energy, recovery and muscular repair, and predisposition to injuries, as previously shown [21,22,23,24]. Although genetics in sport has been deeply studied, only six single nucleotide polymorphisms (SNPs) have been implicated in these factors, such as I/D (rs4646994) in angiotensin-converting enzyme (*ACE*) involved in hypertension, causing, among others, cardiovascular cerebrovascular diseases [25], chronic obstructive pulmonary disease (COPD) [26,27] and idiopathic pulmonary fibrosis [28], alpha-actinin 3 (*ACTN3*) c.1729C>T (rs1815739), involved in cardiometabolic fitness [29], muscle fiber composition, strength, and risk of muscle injury [30,31]. Adenosine monophosphate deaminase 1 (*AMPD1*) c.34C>T (rs17602729) is involved in congestive heart failure [32], muscle metabolism, injury risk, and early fatigue during sports practice [22,23,33]. The muscle-specific creatine kinase (*CKM*) c.*800A>G (rs8111989) polymorphism has been correlated with physical performance and contributes to differences in the maximum oxygen uptake (VO_2_max) responses during training [34]. Finally, myosin light-chain kinase (*MLCK*) c.49C>T and c.37885C>A (rs2700352 and rs28497577) polymorphisms might predispose some individuals to higher values of muscle pain during exercise [35]. However, it has not yet been demonstrated that these genetic markers could be related to the response to aerobic exercise in subjects with diseases that affect the cardiovascular and neuromuscular systems.

Therefore, the aim of this study was to test the response of patients with long COVID through a physical therapy strategy involving the Nordic walking program and how several genetic polymorphisms involved in muscle performance influence physical capabilities. We hypothesize that genetics may play a role in improving the physical abilities and oxidative capacity of long COVID syndrome patients.

## 2. Results

The patients’ characteristics are shown in Table 1.

The polymorphisms analyzed met the Hardy–Weinberg equilibrium in all genes selected (HWE) (all *p* > 0.050) (Table 2).

The genotype frequencies for the six polymorphisms in the patients’ cohort are shown in Table 3.

### 2.1. ACE

**Figure 1:**
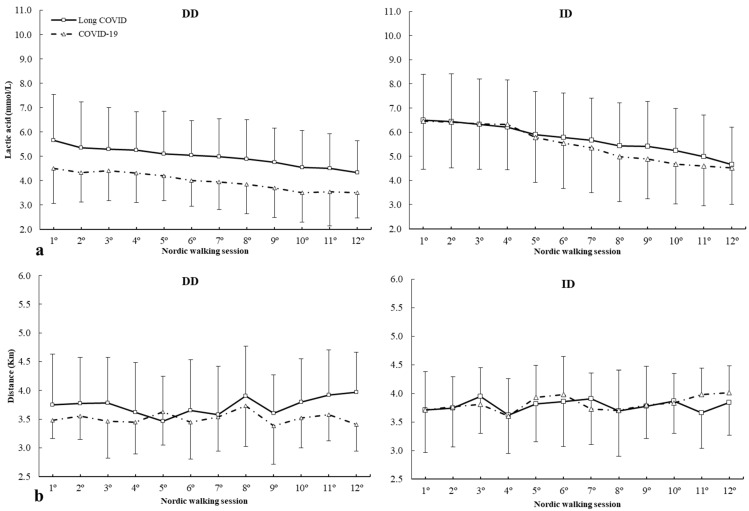
(**a**) Lactic acid and (**b**) distance covered during Nordic walking sessions in patients with different *ACE* ID genotypes.

For I/D (rs4646994) polymorphism, there was effect of lactic acid (F = 2.942, *p* = 0.019), but no main effects of group (F = 0.853, *p* = 0.682) and genotype (F = 2.216, *p* = 0.092), lactic acid × group (F = 1.634, *p* = 0.094), lactic acid × genotype (F = 1.319, *p* = 0.212), and lactic acid × group × genotype interactions (F = 1.897, *p* = 0.106). The posthoc analysis did not reveal any statistically significant effect of lactic acid during any Nordic walking session in all genotypes (all *p* > 0.050) (Figure 1a). For distance (Figure 1b), there were no main effects of genotype (F = 1.451, *p* = 0.471), distance covered (F = 1.036, *p* = 0.446), groups (F = 0.742, *p* = 0.692), and interactions between variables. The posthoc analysis did not reveal any statistically significant effect of distance covered during any Nordic walking session in all genotypes (all *p* > 0.050) (Figure 1b).

### 2.2. ACTN3

**Figure 2:**
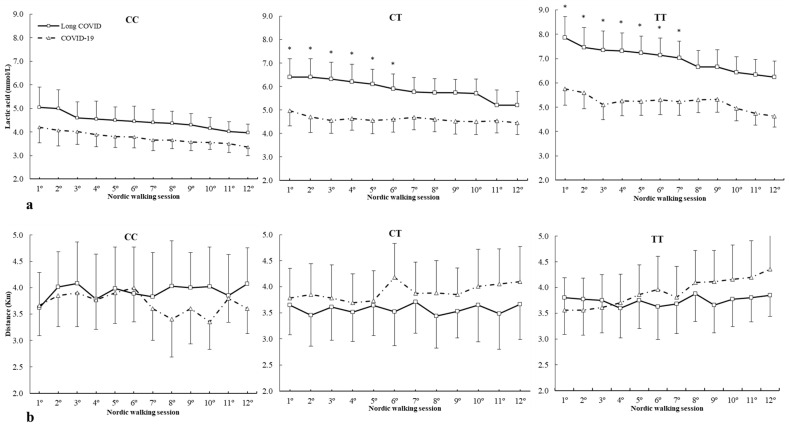
(**a**) Lactic acid and (**b**) distance covered during Nordic walking sessions in patients with different *ACTN3* c.1729C>T genotypes. * COVID-19 is different from long COVID for the same Nordic walking session at *p* < 0.050. mmol/L, millimole/liter; Km, kilometer.

Figure 2 depicts lactic acid concentrations and distance covered during all Nordic walking sessions in c.1729C>T (rs1815739) polymorphism. For lactic acid concentrations (Figure 2a), there was no main effect of lactic acid (F = 1.553, *p* = 0.425), group (F = 0.843, *p* = 0.632), and genotype (F = 0.858, *p* = 0.559). However, there were statistically significant interactions in lactic acid × group × genotype (F = 1.702, *p* = 0.028) with no main effect in interactions among these variables during the Nordic walking program. The posthoc analysis revealed that in comparison with COVID-19, the long COVID group increased lactic acid concentrations in 1º to 6º Nordic walking sessions in the CT genotype and 1º to 7º Nordic walking sessions for the TT genotype (all *p* < 0.05) (Figure 2a). For distance (Figure 2b), there was a main effect of distance covered (F = 3.289, *p* = 0.022) with no main effect of group (F = 1.255, *p* = 0.332) and genotype (F = 1.142, *p* = 0.425). There were no statistically significant interactions between these variables on distance during the Nordic walking program. The posthoc analysis did not reveal any statistically significant effect of distance covered during any Nordic walking session in all genotypes (all *p* > 0.050) (Figure 2b).

### 2.3. AMPD1

**Figure 3:**
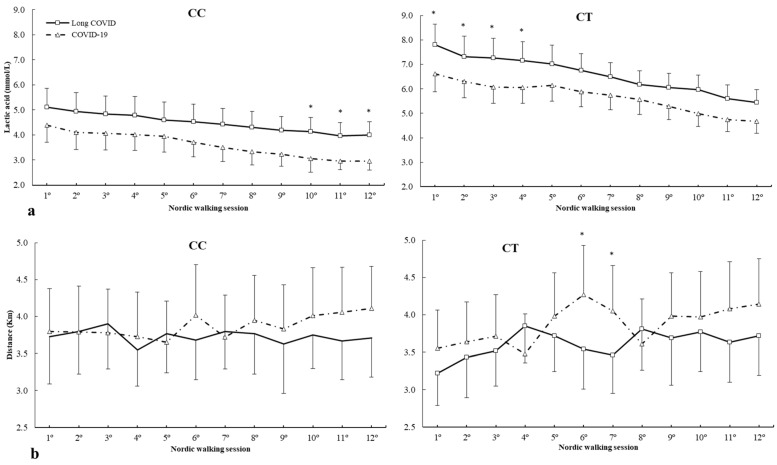
(**a**) Lactic acid and (**b**) distance covered during Nordic walking sessions in patients with different *AMPD1* c.34C>T genotypes. * COVID-19 is different from long COVID for the same Nordic walking session at *p* < 0.050. mmol/L, millimole/liter; Km, kilometer.

For c.34C>T (rs17602729) polymorphism, there were main effects of lactic acid (F = 8.532, *p* < 0.001), group (F = 4.521, *p* = 0.003) and genotype (F = 6.743, *p* < 0.001). There were main effects of lactic acid × genotype (F = 4.479, *p* < 0.001) and lactic acid × group × genotype interactions (F = 1.838, *p* = 0.048) (Figure 3a). The posthoc analysis revealed that, in comparison with COVID-19, the long COVID group increased lactic acid concentrations from 10º to 12º Nordic walking sessions in the CC genotype and 1º to 4º Nordic walking sessions for the CT genotype (all *p* < 0.050) (Figure 3a). For distance, there was a main effect of distance covered (F = 2.889, *p* = 0.033), and there was no main effect of group (F = 1.674, *p* = 0.284) or genotype (F = 3.214, *p* = 0.066) and interactions between variables. The posthoc analysis revealed that, in comparison to long COVID, the COVID-19 patients increased the distance covered in 6º and 7º Nordic walking sessions in the CT genotype (all *p* < 0.050) (Figure 3b).

### 2.4. CKM

**Figure 4:**
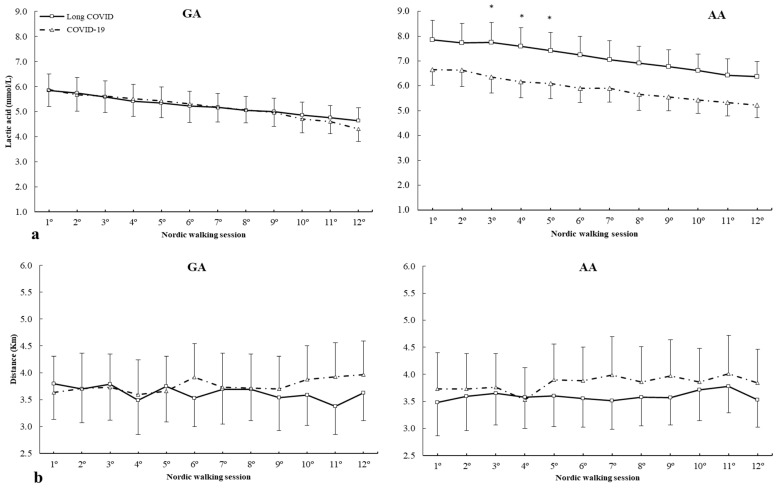
(**a**) Lactic acid and (**b**) distance covered during Nordic walking sessions in patients with different *CKM* c.*800A>G genotypes. * COVID-19 is different from long COVID for the same Nordic walking session at *p* < 0.050. mmol/L, millimole/liter; Km, kilometer.

Figure 4 depicts lactic acid concentrations and distance covered during all Nordic walking sessions in c.*800A>G (rs8111989) polymorphism. For lactic acid concentrations (Figure 4a), there was a main effect of lactic acid (F = 2.852, *p* = 0.041) and genotype (F = 3.024, *p* = 0.032) with no main effect of group (F = 1.174, *p* = 0.418) and interactions between variables. The posthoc analysis revealed that, in comparison with COVID-19, the long COVID group increased lactic acid concentrations from 3º to 5º Nordic walking sessions in the AA genotype (all *p* < 0.050) (Figure 4a). For distance, there were no main effects of distance covered (F = 1.597, *p* = 0.369), group (F = 1.726, *p* = 0.199) or genotype (F = 1.097, *p* = 0.451) and interactions between variables (Figure 4b).

### 2.5. MLCK

**Figure 5:**
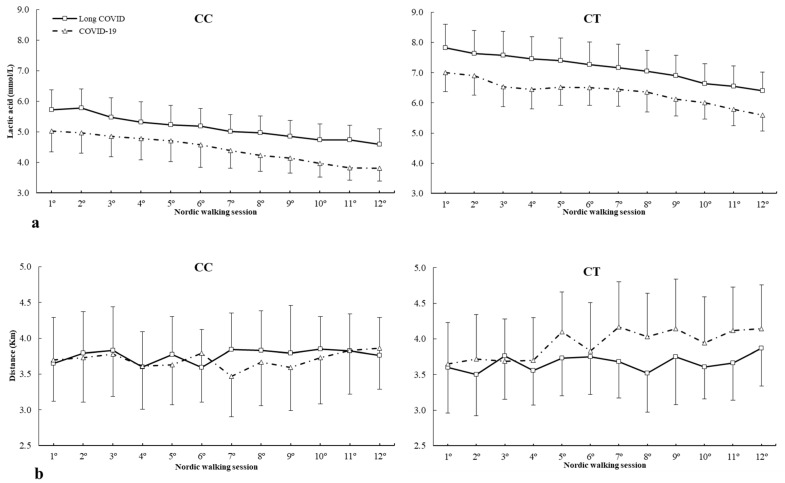
(**a**) Lactic acid and (**b**) distance covered during Nordic walking sessions in patients with different *MLCK* c.49C>T genotypes. mmol/L, millimole/liter; Km, kilometer.

For c.49C>T (rs2700352) polymorphism, there was a main effect of lactic acid (F = 5.673, *p* < 0.001) with no main effects of group (F = 1.132, *p* = 0.472) or genotype (F = 1.942, *p* = 0.117) and interactions between variables (Figure 5a). For distance, there were no main effects of distance covered (F = 2.453, *p* = 0.069), group (F = 2.174, *p* = 0.105) or genotype (F = 0.852, *p* = 0.592) and interactions between variables (Figure 5b).

**Figure 6:**
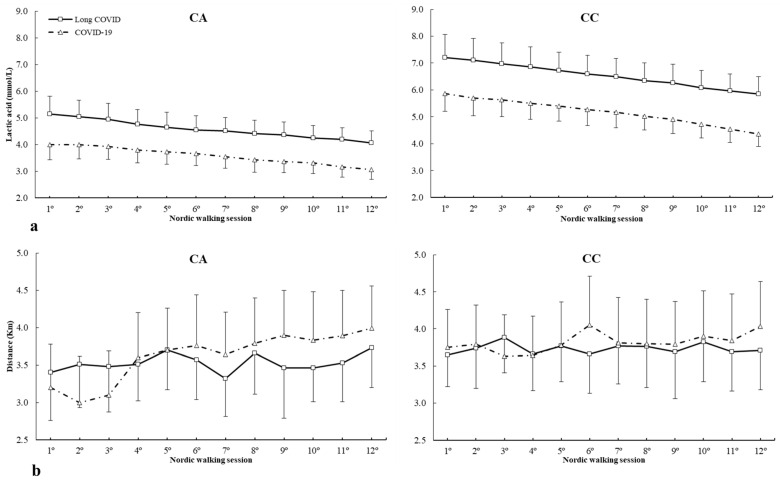
(**a**) Lactic acid and (**b**) distance covered during Nordic walking sessions in patients with different *MLCK* c.37885C>A genotypes. mmol/L, millimole/liter; Km, kilometer.

For MLVCK c.37885C>A (rs28497577) polymorphism, there was a main effect of lactic acid (F = 6.363, *p* < 0.001) with no main effects of group (F = 1.378, *p* = 0.338) or genotype (F = 2.358, *p* = 0.097) and interactions between variables (Figure 6a). For distance, there was a main effect of distance covered (F = 2.841, *p* = 0.035), and there was no main effect of group (F = 0.986, *p* = 0.508) or genotype (F = 0.752, *p* = 0.689) and interactions between variables (Figure 6b).

## 3. Discussion

This nonrandomized controlled pilot study provides relevant information about the influence of several polymorphisms on the benefits derived from physical activity on lactic acid concentrations and physical performance during aerobic exercise programs. Overall, the results of the present study support previous scientific evidence on therapeutic exercise as a strategy to improve physical capacities and decrease fatigue during exercise in patients who have suffered from COVID-19 and present with long COVID syndrome [38,39]. But more importantly, we report, for the first time, the influence of polymorphisms previously implicated in muscle performance on the effect of exercise therapeutics in understanding the impairment of oxidative pathways in patients with long COVID syndrome during aerobic exercise. Taken together, the results of the present study suggest that a Nordic walking program in patients who suffered mild COVID-19, significantly decreases lactic acid concentrations during submaximal aerobic exercise in individuals with genotypes CT and TT in *ACTN3* c.1729C>T, CC and CT in *AMPD1* c.34C>T and AA in *CKM* c.*800A>G polymorphisms. Further studies must be devoted to determining physiological mechanisms explaining the interindividual variability in response to therapeutic exercise context in long COVID patients.

Previous studies have reported the effects of physical therapy in improving the quality of life of patients with long COVID syndrome, focusing on aerobic exercise to improve lung and cardiovascular functions in addition to other symptoms, such as dyspnea, fatigue, and quality of life [5,40,41,42]. The changes observed with an aerobic exercise program suggest an effect of metabolic pathways in shifting the substrates for energy supply during low-to-moderate-intensity aerobic exercise toward a greater reliance on fat rather than carbohydrate and lactic acid production. This effect induced by the enhancement of oxidative pathways may be considered a potential advantage for those individuals with long COVID syndrome who present with fatigue and dyspnea and aid their treatment for improved quality of life. Furthermore, these metabolic effects are in addition to other benefits associated with physical therapy strategy in long COVID syndrome, such as reduced fatigue [42] and increased muscle oxygen saturation [43]. Within physical therapy strategies, research has presented the 6-min walking test (6 MWT) as one of the most effective for these patients due to the physical complications they present and how it helps them to achieve improvements in their physical abilities. Typically, the duration of the program is approximately 12 weeks to evidence the effects [15,44,45].

Nordic walking can be a full-body or alternating aerobic/anaerobic discipline [46,47]. Several studies have shown that improvements in functional capacity, cardiorespiratory fitness, and fitness are achieved with a 12-week Nordic walking protocol in older adults [48,49,50,51]. The current investigation has used Nordic walking therapy for these previously described implications and has shown metabolic-like benefits to decrease lactic acid concentrations in subjects who have previously suffered COVID-19 during the 12 weeks, being the first time it has been demonstrated as physical therapy for the improvement of aerobic capacities in subjects with long COVID syndrome and subjects who have suffered COVID-19, showing the genetics of muscle performance as a tool to personalize and optimize these subjects suffering from this syndrome.

Lactic acid and anaerobic pathways are concepts related to energy production during intense, high-intensity physical exercise. These processes are part of the way the human body obtains energy when it cannot provide sufficient oxygen to sustain regular aerobic metabolism [52]. In some diseases, it has been previously reported that lactic acid concentrations are elevated during exercise due to impaired aerobic metabolism, such as in diseases affecting lung and cardiorespiratory function, like COVID-19 [53]. This research shows that patients with long COVID syndrome have a very high lactic acid threshold, is higher than subjects who have suffered from COVID-19, which could be the cause of these affections on the aerobic pathways in sports practice and could trigger these symptoms so characteristic in this cohort of patients, presenting the need for future research that could corroborate the results presented for the first time in this study.

Genetics has been presented as one of the interindividual factors in predisposing physical abilities and susceptibility to disease [54,55]. Among these genetic markers, several have been shown to be relevant to muscle performance and metabolism [56].

*ACE* I/D (rs4646994) polymorphism became the first genetic element shown to impact human physical performance substantially [57]. Although the involvement of the *ACE* gene in performance and sports injuries has been shown to date, its influence on lactic acid in sports practice is not known, confirming the results shown in this pilot study, which has shown no differences in the different genotypes in the *ACE* gene.

c.1729C>T (rs1815739) polymorphism in *ACTN3* gene is another target widely studied in sports performance [31,58,59]. In this case, the C allele is related to the proportion of fast muscle fibers (type I) and the T allele to slow muscle fibers (type II) [30]. This muscle fiber composition may have an implication in the patients’ response to the Nordic walking program, with the T allele carriers showing statistical differences between the two groups in the first aerobic exercise strategy (Figure 2). These data suggest that patients who did not have long COVID syndrome responded better to the physical exercise strategy in the early phases with lower lactic acid concentration than subjects with long COVID. All subjects decreased lactic acid concentrations with Nordic walking sessions, which will need to be confirmed in future studies. The different genotypes in *ACTN3* did not differ in patient performance in terms of distances covered during the entire Nordic walking program between subjects with long COVID syndrome and those with nonlong COVID. However, the different genotypes in *ACTN3* did not differ in patient performance in terms of distances covered during the entire Nordic walking program between subjects with long COVID syndrome and those without long COVID.

*AMPD1* c.34C>T (rs17602729) polymorphism has previously been linked to athlete status due to its relationship with muscle metabolism [20]. The T allele has been shown to cause early fatigue, cramps and muscle injuries in sports [60]. In the current study, it has been found that the T allele is associated with a higher lactic acid concentration in patients with long COVID syndrome with physical therapy strategy in the first sessions, an aspect that could be related to the fatigue of patients in this group, which can be overcome with the passage of aerobic exercise sessions, an aspect with clinical relevance, while participants with CC genotype have higher lactate concentration in Long COVID syndrome at the end of the program compared to patients who only suffered COVID-19. The program decreases lactic acid concentrations in all study subjects, being higher in those with long COVID, which may be related to lower effectiveness in muscle metabolism, which may be affected in all genotypes.

Muscle damage is an important aspect in COVID-19 patients due to the muscle weakness and severe fatigue caused by the disease and related to different catabolites, such as creatine kinase. In this respect, the c*800A>G (rs8111989) polymorphism of the *CKM*, some patients may experience persistent fatigue, shortness of breath or other symptoms that limit their ability to exercise and may be uniquely associated with the the AA genotype regarding lactic acid in long COVID syndrome subjects.

Aside from the strengths presented, this study has several limitations that should be discussed to enhance the applicability of this study to real therapeutic exercise strategy contexts: (i) Small sample size; the study only included 29 patients. This small number makes it hard to generalize the results to a larger population. (ii) No randomization; patients were not randomly assigned to groups. This can introduce bias, meaning the groups may not be comparable. (iii) Short duration; the study only lasted 12 weeks. This short period may not be enough to see long-term effects. (iv) Single location; all participants were from the same area. This limits the study’s ability to apply its findings to different regions or populations. (v) Selection bias; participants volunteered to join the study, which might mean they are different from the general population (e.g., more motivated or healthier). (vi) Confounding variables; potential confounding variables, such as differences in baseline fitness levels or other lifestyle factors between participants, were not controlled for. (vii) Cardiovascular assessment; future studies should include comprehensive cardiovascular evaluations and subjective exertion scale to understand better the impact of aerobic exercise on heart health in long COVID patients. (viii) Power analysis; this pilot study did not present power of analysis, and future research should include power analysis to ensure that the sample size is adequate to detect significant effects. (ix) Monitored diet; the diet of participants was not specifically monitored or controlled during the study. Future studies should consider dietary assessments or controls to account for nutritional influences on lactate levels and exercise performance. (x) Cardiopulmonary exercise testing (CPET); no patient underwent a CPET to determine their VO_2_max before and after the Nordic walking program. (xi) Limited genetic analysis; the study only looked at a few genetic markers. There might be other important genetic factors not considered, like Insulin-like growth factor I (*IGF-1*), interleukins 6 and 15 (*IL6*, *IL16*), nitric oxide synthase 3 (NOS3), peroxisome proliferator-activated receptor alpha (*PPARA*) and vitamin D receptor (*VDR*) implied in inflammation, muscle disorders, and peripheral vasodilatation during physical activity [21]. These limitations suggest that future research with a larger, more diverse sample, randomization, and longer follow-up is needed.

In clinical practice, health professionals could prescribe exercise to patients with long COVID syndrome. However, patients often experience increased fatigue and worsening symptoms after exercising. Some studies have shown a link between exercise intolerance and dysfunction in the oxygen extraction system in long COVID patients [61]. It is crucial to determine the molecular basis of this fatigue, including lactate levels generated by activity, to prescribe and monitor exercise properly. This will benefit both patients, who can take an active role in self-dosage, and professionals, who can be more confident in their prescriptions based on individual lactate generation and genetic polymorphisms. The better we understand the mechanisms underlying fatigue in these patients, the better we can tailor exercise sessions, including the type, duration, and progression of exercise.

Identifying potential polymorphisms could be valuable when scheduling and prescribing therapeutic exercise to improve physical capacity in long COVID patients. Therefore, the intensity and duration of future Nordic walking programs for this population should take the findings of current research into account, aiming to individualize interventions by considering genetic factors and physical exertion capacity.

## 4. Materials and Methods

### 4.1. Study Design

A two-arm, nonrandomized, controlled pilot study was conducted to determine physiological and genetic responses to aerobic physical exercise strategy through a Nordic walking program by measuring lactate concentration in long COVID syndrome and COVID-19 groups.

### 4.2. Patients

Patients in the long COVID group were recruited by mass mailing to their AMACOP (Asociación Madrileña de Pacientes con Covid Persistente, Madrid, Spain) e-mail accounts. Finally, the sample was divided into a nonrandomized intervention group (long COVID) (n = 16) and a healthy control (COVID-19) group (n = 13), as previously shown [62]. The healthy control group was recruited through advertisements and flyers in the Madrid region. The cases of comorbidities presented in the long COVID group were three subjects with hypertension and two with diabetes mellitus (Table 1).

Inclusion criteria were (i) people between 18 and 65 years old. Long COVID has also been reported in children [63], but this pilot study was focused on the adult population, which ranges from 18 to 65 years old. The participants were close to the age corresponding to the mean age defined by the long COVID patient type [64], (ii) with a complete COVID-19 vaccination schedule, (iii) in the case of the experimental group, they had to provide a documented diagnosis and have persistent long COVID symptoms for at least one year and diagnosed by long COVID syndrome patients of similar age and anthropometric characteristics, who presented symptomatology at the beginning of the study, and voluntary healthy controls were recruited and, (iv) all long COVID patients presented common symptoms as fatigue, dyspnea, cough, and chest pain [65] at the time of recruitment. Exclusion criteria were (i) the presence of comorbidities of musculoskeletal nature that contraindicated the practice of the proposed exercises, (ii) the presence of uncontrolled cardiovascular or respiratory diseases, (iii) the presence of disabling neurological diseases that significantly interfere with the practice of the proposed exercise, (iv) severe anemia or other comorbidities that significantly interfere with the proposed exercise, (e) diagnosis or symptoms of dysautonomia, (v) the presence of high levels of fatigue, (vi) desaturations during the practice test that imply a need for exercise oxygen supply, and (vii) ≥3% effort desaturation during exercise.

Patients signed an informed written consent form to participate in the investigation. The study was registered at the Registry of Clinical Trials (NCT05453188 on 14 July 2022), and ethical approval was obtained by the Ethics Committee of Hospital Universitario Fundación Alcorcón (Ethical Approval Code 21/175). The study was conducted in accordance with the Declaration of Helsinki of 1964 (last update 2013).

### 4.3. Desoxyribonucleic Acid (DNA) Sample Collecting and Genotyping

The samples were collected with SARSTED swabs by buccal smear and refrigerated until genotyping.

DNA extraction from the swabs was carried out in the VIVOLabs laboratory (Madrid, Spain) by automatic extraction in QIACube equipment (QIAGEN, Venlo, Holland), yielding a DNA concentration of 25–40 ng/mL, which was kept in a solution in a volume of 100 μL at −20 °C until genotyping.

*ACE* I/D (rs4646994), *ACTN3* c.1729C>T (rs1815739), *AMPD1* c.34C>T (rs17602729), *CKM* c.*800A>G (rs8111989) and *MLCK* c.49C>T (rs2700352) and c.37885C>A (rs28497577) polymorphisms were genotyped by using Single Nucleotide Primer Extension (SNPE) with the SNaPshot Multiplex Kit (Thermo Fisher Scientific, Waltham, MA, USA), with analysis of the reaction result by capillary electrophoresis fragments, in an ABI3500 unit (Applied Biosystems, Foster City, CA, USA) with bioinformatic analysis performed by GeneMapper 5.0 software (Applied Biosystems, CA, USA).

### 4.4. Nordic Walking Program

The long COVID and COVID-19 groups conducted the Nordic walking sessions for 90 min (warm-up and teaching the technique, exercise, and cool-down) conducted once a week for 12 weeks.

At the beginning of each Nordic walking session, atmospheric humidity and temperature data were recorded. All patients performed a protocol of 45 min of Nordic walking each session for aerobic response, as previously reported [66]. After the session, the distance covered by each patient was recorded, and lactate levels were measured during the first minute immediately after the end of the session. Patients were asked to start Nordic walking in a staggered manner to facilitate immediate lactate measurement. They started walking, leaving one minute between the start of each participant.

Nordic walking sessions were conducted at a moderate-to-vigorous intensity, as previously described [48], completed outdoors weekly from February to May 2023 in Casa de Campo, Madrid, Spain. The instructor started by designing a route without a slope, which was the same during the three months of the intervention.

Instructions were given to all participants during the development of the Nordic walking sessions (i.e., to complete the time of each session, to walk on flat ground for 45 min but they could regulate the pace and the distance, which did not have to be the same from one session to the next. They also had to communicate to the health professionals present in every session if they felt dyspnea, perceived as “they were gasping for breath when they tried to speak”. In addition, all participants were accompanied in all sessions by a nurse specialized in cardiovascular care and two physiotherapists with experience in therapeutic exercise. Patients should not be desaturated during exercise (no more than 3%), and their heart rate values during exercise were taken. Finally, all participants were required to tell the researchers if they felt long-exertional fatigue immediately after the session or during the following 48 h.

### 4.5. Sample Collection

Lactate samples were collected after multiple 45-min Nordic walking sessions once per week for 12 weeks. A nurse took a blood sample (0.3 microliters) from the participants by capillary puncturing the index finger of the hand in order to conduct blood lactate concentration data immediately after physical activity (within the first minute) using the Arkrag lactate Pro2 meter.

### 4.6. Outcome Measures

The distance covered by the participants in all Nordic walking sessions was measured by POLAR Ignite 2 device (Polar Electro, Kempele, Finland).

### 4.7. Statistical Analysis

Analysis was performed using the Statistical Package for the Social Sciences (SPSS), v.21.0 for Windows (IBM Corp. Released 2012. IBM SPSS Statistics for Windows, Version 21.0. Armonk, NY: IBM Corp., Armonk, NY, USA).

Demographical data are presented using chi-square for qualitative variables and paired *t*-test for quantitative variables. Data are presented as mean ± standard deviation (SD) for each genotype. All data were checked for normality and sphericity by using the Shapiro-Wilk test. As all variables presented a normal distribution, parametric statistics were employed to determine differences in response to lactate and distance depending on polymorphisms. Disequilibria of SNPs were estimated using the Hardy–Weinberg Equilibrium (HWE) followed by the approach by Weir and Cockerham [67]. A three-way analysis of variance (ANOVA; 2 × 3 × 12, corresponding to group × genotype × Nordic walking sessions) was used to compare lactate levels and distance covered. When a significant F value was obtained for any main effect or interaction, a least significant difference (LSD) posthoc analysis was performed to determine pairwise differences for the values obtained in the long COVID group vs. the COVID-19 group within each genotype.

The significance level was set at *p* < 0.050.

## 5. Conclusions

The present study is the first to investigate the influence of genetic polymorphisms on performance in subjects with long COVID syndrome. The main findings support that individuals who reported long COVID who presumably were less exercised beforehand appeared to be less able to exercise, based on lactate levels and aerobic physical therapy strategy through a Nordic walking program enhanced physical capabilities during aerobic exercise in long COVID patients with different genotypes in *ACTN3* c.1729C>T and *AMPD1* c.34C>T polymorphisms. Hence, a moderate-intensity aerobic physical therapy strategy improves physical capacity in individuals with COVID-19 and long COVID syndrome supported by genetic information to optimize these strategies for recovery of COVID-19 patients.

## Figures and Tables

**Table 1:** Patients’ characteristics.

		Long COVID (*n* = 16)	COVID-19 (*n* = 13)	*p* Value
Gender	Male, n (%)	1 (6.2)	4 (30.8)	0.082
	Female, n (%)	15 (93.8)	9 (69.2)	
	Age, years (SD)	46.13 (7.91)	46.92 (6.00)	0.766
	Weight, kg (SD)	65.52 (12.52)	63.14 (13.39)	0.363
	Height, cm (SD)	166.23 (8.03)	167.25 (7.99)	0.743
	BMI, Kg/m^2^	23.67 (1.25)	22.62 (1.32)	0.515
Comorbidities	No, n (%)	11 (68.7)	8 (61.5)	0.663
	Yes, n (%)	5 (31.3)	5 (38.5)	
Pulmonary fibrosis after COVID-19	No, n (%)	15 (93.8)	13 (100.0)	0.894
	Yes, n (%)	1 (6.2)	0 (0.0)	

cm, centimeters; kg, kilogram; SD, standard deviation.

**Table 2:** Hardy–Weinberg equilibrium and minor allele frequency (MAF) for selected genes in muscle performance.

Symbol	Gene	dbSNP	Genomic Location	MAF Long COVID Patients	MAF (IBS) *	HWE	FIS
*ACE*	Angiotensin-converting enzyme	rs4646994	17q23.3	40.6% (I)	36.7% (I) **	*p* = 0.463	−0.17
*ACTN3*	Alpha-actinin-3	rs1815739	11q13.2	50.0% (T)	43.9% (T)	*p* = 0.319	−0.24
*AMPD1*	Adenosine monophosphate deaminase 1	rs17602729	1p13.2	18.7% (T)	14.0% (T)	*p* = 0.597	−0.19
*CKM*	Muscle-specific creatine kinase	rs8111989	19q13.32	34.4% (G)	26.6% (G)	*p* = 0.185	−0.31
*MLCK*	Myosin light chain kinase	rs2700352	3q21.1	31.2% (T)	20.1% (T)	*p* = 0.060	−0.49
	Myosin light chain kinase	rs28497577	3q21.1	18.7% (A)	10.3% (A)	*p* = 0.314	−0.33
Overall SNPs						*p* = 0.382	−0.28

IBS, Iberian population in Spain * [36] ** [37]; FIS, inbreeding coefficient; HWE, Hardy–Weinberg equilibrium; MAF, minor allele frequency; SNP, single nucleotide polymorphism.

**Table 3:** Genotype distribution in target polymorphisms on study patients.

Gene	Polymorphism	dbSNP	Genotype	Long COVID (n = 16)	COVID-19 (n = 13)	*p* Value
*ACE*	I/D	rs4646994	DD	5 (31.2)	4 (30.8)	0.975
			ID	9 (56.2)	7 (53.8)	
			II	2 (12.5)	2 (15.4)	
*ACTN3*	c.1729C>T	rs1815739	CC	4 (25.0)	1 (7.7)	0.425
			CT	8 (50.0)	9 (69.2)	
			TT	4 (25.0)	3 (23.1)	
*AMPD1*	c.34C>T	rs17602729	CC	11 (68.8)	8 (61.5)	0.525
			CT	4 (25.0)	5 (38.5)	
			TT	1 (6.2)	0 (0.0)	
*CKM*	c.*800A>G	rs8111989	GG	2 (12.5)	1 (7.7)	0.388
			GA	7 (43.8)	9 (69.2)	
			AA	7 (43.8)	3 (23.1)	
*MLCK*	c.49T>C	rs2700352	CC	9 (56.2)	9 (69.2)	0.257
			CT	4 (25.0)	4 (30.8)	
			TT	3 (18.8)	90 (0.0)	
	c.37885C>A	rs28497577	CA	3 (18.8)	1 (7.7)	0.390
			CC	13 (81.2)	12(92.3)	

SNP, single nucleotide polymorphism.

## Data Availability

The data presented in this study are available upon request from the corresponding author. The data are not publicly available due to legal restrictions.

## References

[1] Callard F., Perego E. (2021). How and why patients made Long Covid. Soc. Sci. Med..

[2] Shah W., Hillman T., Playford E.D., Hishmeh L. (2021). Managing the long term effects of COVID-19: Summary of NICE, SIGN, and RCGP rapid guideline. BMJ.

[3] Burnett D.M., Skinner C.E. (2023). Year in Review: Long COVID and Pulmonary Rehabilitation. Respir. Care.

[4] Besnier F., Bérubé B., Malo J., Gagnon C., Grégoire C.A., Juneau M., Simard F., L’Allier P., Nigam A., Iglésies-Grau J. (2022). Cardiopulmonary Rehabilitation in Long-COVID-19 Patients with Persistent Breathlessness and Fatigue: The COVID-Rehab Study. Int. J. Environ. Res. Public Health.

[5] Nopp S., Moik F., Klok F.A., Gattinger D., Petrovic M., Vonbank K., Koczulla A.R., Ay C., Zwick R.H. (2022). Outpatient Pulmonary Rehabilitation in Patients with Long COVID Improves Exercise Capacity, Functional Status, Dyspnea, Fatigue, and Quality of Life. Respiration.

[6] Dennis A., Wamil M., Alberts J., Oben J., Cuthbertson D.J., Wootton D., Crooks M., Gabbay M., Brady M., Hishmeh L. (2021). Multiorgan impairment in low-risk individuals with post-COVID-19 syndrome: A prospective, community-based study. BMJ Open.

[7] Tziolos N.R., Ioannou P., Baliou S., Kofteridis D.P. (2023). Long COVID-19 Pathophysiology: What Do We Know So Far?. Microorganisms.

[8] Montes-Ibarra M., Oliveira C.L.P., Orsso C.E., Landi F., Marzetti E., Prado C.M. (2022). The Impact of Long COVID-19 on Muscle Health. Clin. Geriatr. Med..

[9] Gérard M., Mahmutovic M., Malgras A., Michot N., Scheyer N., Jaussaud R., Nguyen-Thi P.L., Quilliot D. (2021). Long-Term Evolution of Malnutrition and Loss of Muscle Strength after COVID-19: A Major and Neglected Component of Long COVID-19. Nutrients.

[10] Burgess L.C., Venugopalan L., Badger J., Street T., Alon G., Jarvis J.C., Wainwright T.W., Everington T., Taylor P., Swain I.D. (2021). Effect of neuromuscular electrical stimulation on the recovery of people with COVID-19 admitted to the intensive care unit: A narrative review. J. Rehabil. Med..

[11] Hashmi M.D., Alnababteh M., Vedantam K., Alunikummannil J., Oweis E.S., Shorr A.F. (2020). Assessing the need for transfer to the intensive care unit for Coronavirus-19 disease: Epidemiology and risk factors. Respir. Med..

[12] Morrow A., Gray S.R., Bayes H.K., Sykes R., McGarry E., Anderson D., Boiskin D., Burke C., Cleland J.G.F., Goodyear C. (2022). Prevention and early treatment of the long-term physical effects of COVID-19 in adults: Design of a randomised controlled trial of resistance exercise-CISCO-21. Trials.

[13] Jimeno-Almazán A., Franco-López F., Buendía-Romero Á., Martínez-Cava A., Sánchez-Agar J.A., Sánchez-Alcaraz Martínez B.J., Courel-Ibáñez J., Pallarés J.G. (2022). Rehabilitation for post-COVID-19 condition through a supervised exercise intervention: A randomized controlled trial. Scand. J. Med. Sci. Sports.

[14] Jimeno-Almazán A., Pallarés J.G., Buendía-Romero Á., Martínez-Cava A., Franco-López F., Sánchez-Alcaraz Martínez B.J., Bernal-Morel E., Courel-Ibáñez J. (2021). Post-COVID-19 Syndrome and the Potential Benefits of Exercise. Int. J. Environ. Res. Public Health.

[15] Cano-de-la-Cuerda R., Jiménez-Antona C., Melián-Ortiz A., Molero-Sánchez A., Gil-de Miguel Á., Lizcano-Álvarez Á., Hernández-Barrera V., Varillas-Delgado D., Laguarta-Val S. (2022). Construct Validity and Test-Retest Reliability of a Free Mobile Application to Evaluate Aerobic Capacity and Endurance in Post-COVID-19 Syndrome Patients—A Pilot Study. J. Clin. Med..

[16] Kalil-Filho R., Saretta R., Franci A., Baracioli L.M., Galas F., Gil J.S., Ferino A., Giacovone C., Oliveira I., Souza J. (2023). Post-COVID-19 Cardiopulmonary Symptoms: Predictors and Imaging Features in Patients after Hospital Discharge. Arq. Bras. Cardiol..

[17] Piotrowicz K., Gąsowski J., Michel J.P., Veronese N. (2021). Post-COVID-19 acute sarcopenia: Physiopathology and management. Aging Clin. Exp. Res..

[18] Dotan A., David P., Arnheim D., Shoenfeld Y. (2022). The autonomic aspects of the post-COVID19 syndrome. Autoimmun. Rev..

[19] Colas C., Le Berre Y., Fanget M., Savall A., Killian M., Goujon I., Labeix P., Bayle M., Féasson L., Roche F. (2023). Physical Activity in Long COVID: A Comparative Study of Exercise Rehabilitation Benefits in Patients with Long COVID, Coronary Artery Disease and Fibromyalgia. Int. J. Environ. Res. Public Health.

[20] Varillas-Delgado D., Morencos E., Gutiérrez-Hellín J., Aguilar-Navarro M., Muñoz A., Mendoza Láiz N., Perucho T., Maestro A., Tellería-Orriols J.J. (2022). Genetic profiles to identify talents in elite endurance athletes and professional football players. PLoS ONE.

[21] Hughes D.C., Day S.H., Ahmetov I.I., Williams A.G. (2011). Genetics of muscle strength and power: Polygenic profile similarity limits skeletal muscle performance. J. Sports Sci..

[22] Maestro A., Del Coso J., Aguilar-Navarro M., Gutiérrez-Hellín J., Morencos E., Revuelta G., Ruiz Casares E., Perucho T., Varillas-Delgado D. (2022). Genetic profile in genes associated with muscle injuries and injury etiology in professional soccer players. Front. Genet..

[23] Varillas Delgado D., Tellería Orriols J.J., Monge Martín D., Del Coso J. (2020). Genotype scores in energy and iron-metabolising genes are higher in elite endurance athletes than in nonathlete controls. Appl. Physiol. Nutr. Metab..

[24] Varillas Delgado D., Telleria Orriols J.J., Martin Saborido C. (2019). Liver-Metabolizing Genes and Their Relationship to the Performance of Elite Spanish Male Endurance Athletes; a Prospective Transversal Study. Sports Med. Open.

[25] Vasudeva K., Balyan R., Munshi A. (2020). ACE-Triggered Hypertension Incites Stroke: Genetic, Molecular, and Therapeutic Aspects. Neuromol. Med..

[26] Zhang X., Wang C., Dai H., Lin Y., Zhang J. (2008). Association between angiotensin-converting enzyme gene polymorphisms and exercise performance in patients with COPD. Respirology.

[27] Kang S.W., Kim S.K., Chung J.H., Jung H.J., Kim K.I., Kim J., Ban J.Y. (2016). Genetic Polymorphism of Angiotensin-Converting Enzyme and Chronic Obstructive Pulmonary Disease Risk: An Updated Meta-Analysis. BioMed Res. Int..

[28] Uh S.T., Kim T.H., Shim E.Y., Jang A.S., Park S.W., Park J.S., Park B.L., Choi B.W., Shin H.D., Kim D.S. (2013). Angiotensin-converting enzyme (ACE) gene polymorphisms are associated with idiopathic pulmonary fibrosis. Lung.

[29] Deschamps C.L., Connors K.E., Klein M.S., Johnsen V.L., Shearer J., Vogel H.J., Devaney J.M., Gordish-Dressman H., Many G.M., Barfield W. (2015). The ACTN3 R577X Polymorphism Is Associated with Cardiometabolic Fitness in Healthy Young Adults. PLoS ONE.

[30] Norman B., Esbjörnsson M., Rundqvist H., Österlund T., Glenmark B., Jansson E. (2014). ACTN3 genotype and modulation of skeletal muscle response to exercise in human subjects. J. Appl. Physiol..

[31] Yang N., MacArthur D.G., Gulbin J.P., Hahn A.G., Beggs A.H., Easteal S., North K. (2003). ACTN3 genotype is associated with human elite athletic performance. Am. J. Hum. Genet..

[32] Binkley P.F., Auseon A., Cooke G. (2004). A polymorphism of the gene encoding AMPD1: Clinical impact and proposed mechanisms in congestive heart failure. Congest. Heart Fail..

[33] Maltese P.E., Venturini L., Poplavskaya E., Bertelli M., Cecchin S., Granato M., Nikulina S.Y., Salmina A., Aksyutina N., Capelli E. (2016). Genetic evaluation of AMPD1, CPT2, and PGYM metabolic enzymes in patients with chronic fatigue syndrome. Genet. Mol. Res..

[34] Chen C., Sun Y., Liang H., Yu D., Hu S. (2017). A meta-analysis of the association of CKM gene rs8111989 polymorphism with sport performance. Biol. Sport.

[35] Clarkson P.M., Hoffman E.P., Zambraski E., Gordish-Dressman H., Kearns A., Hubal M., Harmon B., Devaney J.M. (2005). ACTN3 and MLCK genotype associations with exertional muscle damage. J. Appl. Physiol..

[36] Yates A.D., Achuthan P., Akanni W., Allen J., Alvarez-Jarreta J., Amode M.R., Armean I.M., Azov A.G., Bennett R., Bhai J. (2020). Ensembl 2020. Nucleic Acids Res..

[37] Fiuza-Luces C., Ruiz J.R., Rodríguez-Romo G., Santiago C., Gómez-Gallego F., Cano-Nieto A., Garatachea N., Rodríguez-Moreno I., Morán M., Lucia A. (2011). Is the ACE I/D polymorphism associated with extreme longevity? A study on a Spanish cohort. J. Renin Angiotensin Aldosterone Syst..

[38] Fernández-Lázaro D., Santamaría G., Sánchez-Serrano N., Lantarón Caeiro E., Seco-Calvo J. (2022). Efficacy of Therapeutic Exercise in Reversing Decreased Strength, Impaired Respiratory Function, Decreased Physical Fitness, and Decreased Quality of Life Caused by the Post-COVID-19 Syndrome. Viruses.

[39] Rooney S., Webster A., Paul L. (2020). Systematic Review of Changes and Recovery in Physical Function and Fitness After Severe Acute Respiratory Syndrome-Related Coronavirus Infection: Implications for COVID-19 Rehabilitation. Phys. Ther..

[40] Szarvas Z., Fekete M., Horvath R., Shimizu M., Tsuhiya F., Choi H.E., Kup K., Fazekas-Pongor V., Pete K.N., Cserjesi R. (2023). Cardiopulmonary rehabilitation programme improves physical health and quality of life in post-COVID syndrome. Ann. Palliat. Med..

[41] Volckaerts T., Vissers D., Burtin C., Van Meerbeeck X., de Soomer K., Oostveen E., Claes K., Roelant E., Verhaegen I., Thomeer M. (2023). Randomised, controlled, open-label pragmatic trial evaluating changes in functional exercise capacity after primary care PUlmonary REhabilitation in patients with long COVID: Protocol of the PuRe-COVID trial in Belgium. BMJ Open.

[42] Araújo B.T.S., Barros A., Nunes D.T.X., Remígio de Aguiar M.I., Mastroianni V.W., de Souza J.A.F., Fernades J., Campos S.L., Brandão D.C., Dornelas de Andrade A. (2023). Effects of continuous aerobic training associated with resistance training on maximal and submaximal exercise tolerance, fatigue, and quality of life of patients post-COVID-19. Physiother. Res. Int..

[43] Binetti J., Real M., Renzulli M., Bertran L., Riesco D., Perpiñan C., Mohedano A., Segundo R.S., Ortiz M., Porras J.A. (2023). Clinical and Biomarker Profile Responses to Rehabilitation Treatment in Patients with Long COVID Characterized by Chronic Fatigue. Viruses.

[44] Omar A., Ferreira A.S., Hegazy F.A., Alaparthi G.K. (2023). Cardiorespiratory Response to Six-Minute Step Test in Post COVID-19 Patients—A Cross Sectional Study. Healthcare.

[45] Eksombatchai D., Wongsinin T., Phongnarudech T., Thammavaranucupt K., Amornputtisathaporn N., Sungkanuparph S. (2021). Pulmonary function and six-minute-walk test in patients after recovery from COVID-19: A prospective cohort study. PLoS ONE.

[46] Bullo V., Gobbo S., Vendramin B., Duregon F., Cugusi L., Di Blasio A., Bocalini D.S., Zaccaria M., Bergamin M., Ermolao A. (2018). Nordic Walking Can Be Incorporated in the Exercise Prescription to Increase Aerobic Capacity, Strength, and Quality of Life for Elderly: A Systematic Review and Meta-Analysis. Rejuvenation Res..

[47] Kettinen J., Tikkanen H., Venojärvi M. (2023). Comparative effectiveness of playing golf to Nordic walking and walking on acute physiological effects on cardiometabolic markers in healthy older adults: A randomised cross-over study. BMJ Open Sport. Exerc. Med..

[48] Reed J.L., Terada T., Cotie L.M., Tulloch H.E., Leenen F.H., Mistura M., Hans H., Wang H.W., Vidal-Almela S., Reid R.D. (2022). The effects of high-intensity interval training, Nordic walking and moderate-to-vigorous intensity continuous training on functional capacity, depression and quality of life in patients with coronary artery disease enrolled in cardiac rehabilitation: A randomized controlled trial (CRX study). Prog. Cardiovasc. Dis..

[49] Cokorilo N., Ruiz-Montero P.J., González-Fernández F.T., Martín-Moya R. (2022). An Intervention of 12 Weeks of Nordic Walking and Recreational Walking to Improve Cardiorespiratory Capacity and Fitness in Older Adult Women. J. Clin. Med..

[50] Morat T., Krueger J., Gaedtke A., Preuss M., Latsch J., Predel H.G. (2017). Effects of 12 weeks of Nordic Walking and XCO Walking training on the endurance capacity of older adults. Eur. Rev. Aging Phys. Act..

[51] Ochman M., Maruszewski M., Latos M., Jastrzębski D., Wojarski J., Karolak W., Przybyłowski P., Zeglen S. (2018). Nordic Walking in Pulmonary Rehabilitation of Patients Referred for Lung Transplantation. Transplant. Proc..

[52] Brooks G.A. (2020). Lactate as a fulcrum of metabolism. Redox Biol..

[53] Gupta G.S. (2022). The Lactate and the Lactate Dehydrogenase in Inflammatory Diseases and Major Risk Factors in COVID-19 Patients. Inflammation.

[54] Bouchard C., Rankinen T., Timmons J.A. (2011). Genomics and genetics in the biology of adaptation to exercise. Compr. Physiol..

[55] Bouchard C. (2012). Genomic predictors of trainability. Exp. Physiol..

[56] Varillas-Delgado D., Del Coso J., Gutiérrez-Hellín J., Aguilar-Navarro M., Muñoz A., Maestro A., Morencos E. (2022). Genetics and sports performance: The present and future in the identification of talent for sports based on DNA testing. Eur. J. Appl. Physiol..

[57] Puthucheary Z., Skipworth J.R., Rawal J., Loosemore M., Van Someren K., Montgomery H.E. (2011). The ACE gene and human performance: 12 years on. Sports Med..

[58] Yang N., Garton F., North K. (2009). alpha-actinin-3 and performance. Med. Sport. Sci..

[59] Guth L.M., Roth S.M. (2013). Genetic influence on athletic performance. Curr. Opin. Pediatr..

[60] Fedotovskaya O.N., Danilova A.A., Akhmetov I.I. (2013). Effect of AMPD1 gene polymorphism on muscle activity in humans. Bull. Exp. Biol. Med..

[61] Norweg A., Yao L., Barbuto S., Nordvig A.S., Tarpey T., Collins E., Whiteson J., Sweeney G., Haas F., Leddy J. (2023). Exercise intolerance associated with impaired oxygen extraction in patients with long COVID. Respir. Physiol. Neurobiol..

[62] Varillas-Delgado D., Jimenez-Antona C., Lizcano-Alvarez A., Cano-de-la-Cuerda R., Molero-Sanchez A., Laguarta-Val S. (2023). Predictive Factors and ACE-2 Gene Polymorphisms in Susceptibility to Long COVID-19 Syndrome. Int. J. Mol. Sci..

[63] Demirbuğa A., Hançerli Törün S., Kaba Ö., Dede E., Mete Atasever N., Eryılmaz C.C., Okay N.S., Somer A. (2023). Long COVID in Children: A Pediatric Center Experience. Mikrobiyol. Bul..

[64] López-Sampalo A., Bernal-López M.R., Gómez-Huelgas R. (2022). Persistent COVID-19 syndrome. A narrative review. Rev. Clin. Esp..

[65] Yong S.J. (2021). Long COVID or post-COVID-19 syndrome: Putative pathophysiology, risk factors, and treatments. Infect. Dis..

[66] Liu Y., Xie W., Li J., Ossowski Z. (2023). Effects of aerobic exercise on metabolic indicators and physical performance in adult NAFLD patients: A systematic review and network meta-analysis. Medicine.

[67] Weir B.S., Hill W.G. (2002). Estimating F-statistics. Annu. Rev. Genet..

